# RNA-associated glycoconjugates highlight potential ambiguities in glycoRNA analysis

**DOI:** 10.1038/s12276-025-01585-z

**Published:** 2025-11-15

**Authors:** Sungchul Kim, Zeshi Li, Yong-geun Choi, Kirsten Janssen, Jan-Willem H. Langenbach, Daan J. van den Brink, Christian Büll, Bhagyashree S. Joshi, Adam Pomorski, Vered Raz, Marvin E. Tanenbaum, Pascal Miesen, Chirlmin Joo

**Affiliations:** 1 Center for RNA Research, Institute for Basic Science, Seoul, Republic of Korea; 2 Department of Life Sciences, Pohang University of Science and Technology, Pohang, Republic of Korea; 3 Institute of Convergence Science, Yonsei University, Seoul, Republic of Korea; 4 Division of Chemical Biology and Drug Discovery, Utrecht Institute for Pharmaceutical Sciences, Utrecht University, Utrecht, The Netherlands; 5 Department of Medical Microbiology, Radboud University Medical Center, Nijmegen, The Netherlands; 6 Department of Biomolecular Chemistry, Institute for Molecules and Materials, Radboud University, Nijmegen, The Netherlands; 7 Department of BioNanoScience, Kavli Institute of Nanoscience Delft, Delft University of Technology, Delft, The Netherlands; 8 Department of Chemical Biology, Faculty of Biotechnology, University of Wrocław, Wrocław, Poland; 9 Human Genetics department, Leiden University Medical Centre, Leiden, The Netherlands; 10 Oncode Institute, Hubrecht Institute–KNAW and University Medical Center Utrecht, Utrecht, The Netherlands; 11 Department of Physics, Ewha Womans University, Seoul, Republic of Korea; 12 Present Address: Whitehead Institute for Biomedical Research, Cambridge, MA USA

**Keywords:** Glycobiology, Biochemistry

## Abstract

A recent ground-breaking study suggested that small RNA from mammalian cells can undergo N-glycan modifications (termed glycoRNA)^1^. The discovery relied upon a metabolic glycan labeling strategy in combination with commonly used phase-separation-based RNA isolation. Following the reported procedure, here we likewise identify an N-glycosylated species in the RNA fraction. However, our results suggest that the reported RNase sensitivity of the glycosylated species depends on the specific RNA purification method. This suggests the possibility of copurifying unexpected RNase-insensitive N-glycoconjugates during glycoRNA isolation. The co-existence of two independent, yet highly similar molecular entities, complicates biochemical assays on glycoRNA and calls for more specific approaches for glycoRNA analysis. To address this, we propose a control experiment that can help distinguish genuine glycoRNA species from copurified glycoconjugates.

## Non-RNA glycoconjugates interfere with detection of glycosylated RNA

N-linked glycosylation is a process that modifies proteins, affecting their function and stability. Recently, researchers discovered that small RNA molecules in mammalian cells also have these sugar modifications, called glycoRNA. Researchers found that during RNA extraction, non-RNA molecules with similar sugar modifications, called N-glycoconjugates, are often copurified with RNA. These N-glycoconjugates are resistant to enzymes that typically degrade RNA, suggesting they are not RNA. The study used various methods to isolate and analyze these molecules, including silica columns and chemical labeling techniques. They concluded that previous methods might have misidentified these N-glycoconjugates as glycoRNA due to their similar properties. This finding suggests that more accurate methods are needed to distinguish between true glycoRNA and other sugar-modified molecules. Future research could focus on developing better techniques for studying these complex biomolecules and understanding their roles in cells.

This summary was initially drafted using artificial intelligence, then revised and fact-checked by the author.

## Introduction

N-linked glycosylation is a major post-translational modification that affects folding, stability, and other cellular functions of secretory and membrane-associated proteins^2^. N-glycosylation starts in the endoplasmic reticulum by the assembly of high-mannose glycans and the transfer thereof to nascent peptides. The glycan is then trimmed by ER mannosidases and is further elaborated in the Golgi apparatus, where multiantennary branching and extension, fucosylation, and sialylation are introduced^3^. Expanding the world of N-glycosylation, a recent study reported that specific small noncoding RNA species in mammalian cells are modified with sialylated and fucosylated N-glycans^1^. These molecules, referred to as glycoRNA, were reported to localize to the surface of mammalian cells and were shown to interact with either specific Siglec family receptors or P-selectin^1,4,5^.

Bioorthogonal labeling of glycans present in RNA isolates was a critical step in the discovery of glycoRNA^1^. Carbohydrate analogs or their precursors modified with bioorthogonal chemistry tags, called metabolic chemical reporters (MCRs), are important tools in glycoscience and enable the delineation of the biogenesis and function of glycosylation. MCR, combined with bioorthogonal labeling, is a widely used method due to its simplicity of implementation and the rapidity of the chemical reactions, as well as the bio-compatibility and high specificity in biological environments^6^. MCRs exploit the tolerance of cellular glycan biosynthetic pathways towards unnatural modifications, which are eventually incorporated into glycans^7^. For example, peracetylated *N*-azidoacetyl-mannosamine (Ac_4_ManNAz) has been used as MCRs for sialic acid labeling. Ac_4_ManNAz is converted into azido-sialic acids (SiaNAz) and is normally incorporated at the terminus of glycans^8^. The azide tag in sialoglycans can be conjugated to alkyne-containing molecules, for instance, fluorophores or biotin, via copper-catalyzed azide–alkyne cycloaddition or copper-free strain-promoted alkyne-azide cycloaddition^9,10^. The latter reaction was used to demonstrate the presence of glycoRNA^1^.

A common method to isolate cellular RNA is to use acidic guanidinium-thiocyanate-phenol-chloroform (AGPC) phase partition^1^. This technique relies on chaotropic agents for cell lysis and protein denaturation, followed by phenol-chloroform-based phase separation for isolation of RNA from other cellular components^11,12^. Following alcohol precipitation of the RNA from the AGPC aqueous phase, the sample normally undergoes further clean-ups using proteases and DNases, after which RNA is assumed to have high purity^12^. Silica solid-phase extraction has been a facile complementary method to AGPC-based RNA purification, as it is believed to yield RNA with exquisite purity^13^. Although functionally pure RNA sample can be obtained, both methods rely upon the physical chemical properties of RNA. As a result, biomolecules with similar properties can be co-isolated^14,15^.

Here, we report that non-RNA N-glycoconjugates persist throughout RNA sample processing steps. We have found that these N-glycoconjugates consistently associate with RNA, regardless of extraction methods used, including acidic phenol-chloroform and silica-based column extraction. In commonly used biochemical assays, these N-glycoconjugates exhibit properties difficult to distinguish from what has been described for glycoRNA^1,4,5^, in terms of enzymatic sensitivities and mobility in gel electrophoresis. A key difference between the N-glycoconjugates and glycoRNA is that the former is predicted to be resistant to RNase digestion. However, we found that when a silica column clean-up was performed after RNase treatment—as in all reported procedure for glycoRNA preparation^1,4,5^—the signals for the N-glycoconjugates were lost in fluorescent gels or blots, closely resembling the effect of RNase digestion of glycoRNA. The signals of the N-glycoconjugates could be recovered either by increasing the alcohol concentration in buffers for silica column loading or by adding exogenous RNA before loading into the column. We also demonstrate that the covalent attachment of synthetic glycans to purified RNA does not confer the resistance toward RNase digestion, supporting the non-RNA nature of the N-glycoconjugates. Taken together, our data demonstrate that RNA isolation methods copurify RNase-insensitive N-glycoconjugates that can be misinterpreted as glycoRNA. This finding suggests that the biochemical assays previously used to characterize glycoRNA should not be used as evidence to distinguish between RNA-linked glycans and copurifying glycoconjugates. Our conclusion is based on experiments performed in four laboratories using reagents that were independently purchased.

## Materials and methods

### Mammalian cell culture

**Fig. 1:**
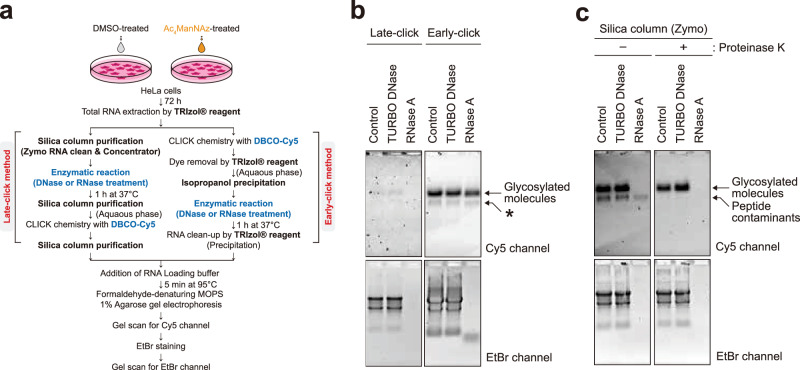
RNase sensitivity of glycosylated molecules depends on the RNA extraction method. **a** A schematic comparison of early click and late-click protocols. **b** Glycan detection by early click and late-click methods. The asterisk indicates the presence of labeled peptide contaminants (see **c**). **c** Glycan visualization with or without proteinase K treatment.

**Fig. 2:**
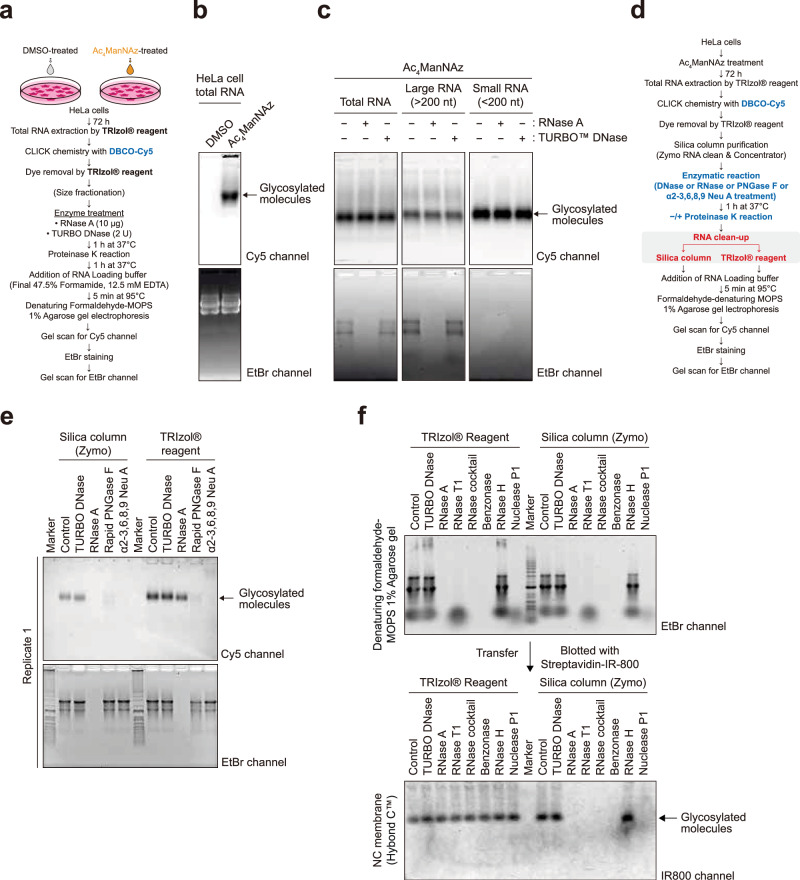
Glycoconjugates copurified with RNA are insensitive to RNase treatment. **a** Schematics of the experimental procedure for labeling and visualization. **b** Glycan detection in Cy5 channel from a denaturing agarose gel without any enzymatic treatment condition. DMSO was used as an Ac_4_ManNAz untreated control. EtBr channel imaged on the gel scanner shows RNAs were intact. **c** Glycan visualization in total RNA from HeLa cells or size-fractionated RNAs. **d** A schematic representation of experiments in Fig. 2e, f. **e** A comparison of RNA clean-up steps between silica column and TRIzol protocols for glycan visualization. DBCO-Cy5-labeled RNA samples were reacted with TURBO DNase or RNase A or Rapid PNGase F or α2-3,6,8,9 neuraminidase A. The data represent one of three replicates. **f** Blotting of biotinylated glycosylated molecules treated with various nucleases using streptavidin-conjugated IR800 dyes on the nitrocellulose membrane. Right: gel image of EtBr-stained RNA samples. Left: fluorescent image using IR800-streptavidin in 800-nm channel.

HeLa cells were cultured at 37 °C and 5% CO_2_ in Dulbecco’s modified Eagle’s medium (DMEM) (Welgene for Figs. 1b, c, 2b, c, e, f, 4b, c and 5b, c and Supplementary Figs. 1, 2b–f, 4a, 5d,e and Gibco for Supplementary Fig. 2e) supplemented with 10% fetal bovine serum (FBS) (Welgene for Figs. 1b, c, 2b, c, e, f, 4b, c and 5b, c and Supplementary Figs. 1, 2b,c,d,f, 4a and 5d,e and Sigma-Aldrich for Supplementary Fig. 2e) and also supplemented with penicillin–streptomycin (Sigma-Aldrich, Supplementary Fig. 2e). The cells were maintained in 100-mm cell culture dishes (Figs. 1b, c, 2b, c, f, 4b, c and 5b, c and Supplementary Figs. 1, 2b,c,d,f, 4a and 5d,e) with 10 ml of culture media or in T75 flasks (Supplementary Fig. 2e). When reaching confluency, cells were split for subculturing. K562 cells (Supplementary Fig. 5b) were cultured at 37 °C and 5% CO_2_ in RPMI medium 1640 supplemented with 10% FBS (Cytiva) and penicillin–streptomycin (Sigma-Aldrich) with shaking at 120 rpm.

### Labeling with MCRs

Stocks of 500 mM *N*-azidoacetylmannosamine-tetraacylated (Ac_4_ManNAz) (Sigma-Aldrich, Figs. 1b, c, 2b, c, e, f, 4b, c and 5b, c and Supplementary Figs. 1, 2b,c,d,f,4a and 5b,d,e) were prepared in sterile dimethyl sulfoxide (DMSO) (Sigma-Aldrich). For metabolic labeling, we treated cells with Ac_4_ManNAz at a final concentration of 100 µM in fresh DMEM supplemented with 10% FBS. After 72 h incubation at 37 °C and 5% CO_2_, the cells were washed with Dulbecco’s phosphate-buffered saline (PBS) (Welgene, Figs. 1b, c, 2b, c, e, f, 4b, c and 5b, c and Supplementary Figs. 1, 2b,c,d,f, 4a and 5b,d,e) twice and stored in −80 °C until total RNA extraction. For experiments shown in Supplementary Fig. 5b, the conditions for metabolic labeling were the same, except for that penicillin–streptomycin was added in the media. For experiments shown in Supplementary Fig. 2e, of 50 mM stocks of Ac_4_ManNAz (custom synthesis by Synvenio) were prepared in sterile DMSO (Merck). The metabolic labeling was done with 100 µM Ac_4_ManNAz in DMEM (Gibco) supplemented with 10% FBS (Sigma-Aldrich) and 1% penicillin–streptomycin. After 48 h at 37 °C and 5% CO_2_, the cells were washed with PBS (in-house preparation) and then followed by total RNA extraction.

### Total RNA extraction with TRIzol reagent

For the experiments shown in Figs. 1b, c, 2b, c, e, f, 4b, c and 5b, c and Supplementary Figs. 1, 2b,c,d,f, 4a and 5d,e, 1 ml of TRIzol reagent (Thermo Fisher Scientific) was added directly onto a washed cell culture dish. The dishes were rocked thoroughly for 10 min at room temperature to lyse and denature all cells. For extracting total RNA from K562 cells (Supplementary Fig. 5b), the cell pellets washed twice with Dulbecco’s PBS were lysed in TRIzol reagent (Thermo Fisher Scientific) with 1 ml for ~10^7^ cells). Homogenized TRIzol-cell mixtures were scraped with cell scraper and then transferred into nuclease-free sterile 1.7 ml microtubes. The tubes were vortexed at least for 5 min until complete homogenization for further denaturation of the intermolecular noncovalent interactions. Phase separation was initiated by adding 200 µl (0.2× volumes) of 100% chloroform (Sigma-Aldrich) into 1-ml TRIzol-dissolved cell mixture and then vortexed thoroughly for complete homogenization. The homogenates were centrifuged at 16,000*g* for 10 min at 4 °C. The upper (aqueous) phase was carefully removed, transferred into a nuclease-free sterile 1.7 ml and then mixed with equal volume of 100% isopropanol (Fisher Scientific) by vortexing. The mixture was centrifuged at 16,000*g* for 30 min at 4 °C, and the supernatant was carefully discarded. The pellet was washed with 1 ml of ice-cold 75% ethanol (Sigma-Aldrich) twice and then dried completely. The RNA pellet was dissolved with Milli-Q (Thermo Fisher Scientific, Figs. 1b, c, 2b, c, f, 4b, c, 5b, c and Supplementary Figs. 1, 2b–f, 4a and 5d,e) H_2_O or Diethyl pyrocarbonate (DEPC)-treated H_2_O (Thermo Fisher Scientific, Supplementary Fig. 5b), and the concentration was measured using a Nanodrop 2000 ultraviolet–visible light spectrophotometer (Thermo Fisher Scientific).

For experiments shown in Supplementary Fig. 2e, 6 ml of TRI Reagent Solution (Thermo Fisher Scientific) were added directly to the washed T75 cell culture flask. The homogenates were vortexed for 1 min at room temperature followed by incubating the samples 1 min at 37 °C. A total of 0.2× volumes of chloroform were added, and phase separation was performed at 16,000*g* for 10 min at room temperature. After adding equal volume of isopropanol, mixtures were incubated for 10 min at 4 °C. The RNA was precipitated at 16,000*g* for 10 min at 4 °C, washed twice with 75% ethanol and dissolved in nuclease-free H_2_O. To obtain highly pure RNA preparations, the isolated RNA was repurified by adding 1 ml of TRI Reagent Solution (Thermo Fisher Scientific) and repeating the isolation procedure described above.

### Copper-free click chemistry and removal of free ligands

Strain-promoted alkyne-azide cycloadditions was performed to probe for azide-containing sialo-conjugates in RNA samples using dibenzocyclooctyne-conjugated cyanine 5 (DBCO-Cy5) (Sigma-Aldrich) dyes or poly-ethylene glycol-spaced dibenzocyclooctyne-biotin (DBCO-biotin, Sigma-Aldrich) as the alkyne-azide cycloaddition. Stocks of 10 mM DBCO-Cy5 or DBCO-biotin were made by dissolving 1 mg of lyophilized DBCO-Cy5 in 82.6 µl or 5 mg of DBCO-biotin in 666.7 µl of DMSO, respectively. A total of 9 µl (typically ~50 µg) of RNA dissolved in H_2_O were mixed at first with 10 µl of home-made 2× dye-free RNA loading buffer (95% formamide, 25 mM ethylenediaminetetraacetic acid, pH 8.0) and added with 1 µL of 10 mM (for final 500 µM) DBCO-Cy5 or DBCO-biotin in a microtube. The samples were incubated at 55 °C for 10 min. The reaction was stopped by adding 1 ml of TRIzol reagent (Thermo Fisher Scientific) and 200 µl of chloroform (Sigma-Aldrich). Alternatively, for experiments performed in Supplementary Fig. 2e, dye removal was achieved by adding 80 µl of DEPC-treated H_2_O, 300 µl of TRIzol LS Reagent (Thermo Fisher Scientific) and 80 µl of chloroform (Merck). The samples were centrifuged at 16,000*g* for 10 min at 4 °C or, for experiments shown in Supplementary Fig. 2e, at 16,000*g* for 5 min at room temperature. The upper (aqueous) phase was carefully removed and transferred into a nuclease-free sterile tube. The samples were mixed with equal volume of 100% isopropanol by vortexing, subsequently centrifuged at 16,000*g* for 30 min at 4 °C, and the supernatant was carefully discarded. The pellet was washed with 1 ml of ice-cold 75% ethanol twice and then dried completely. The RNA pellet was dissolved with Milli-Q H_2_O (Figs. 1b, c, 2b, c, e, f, 4b, c and 5b, c and Supplementary Figs. 1, 2b–f, 4a and 5d,e) or DEPC-treated H_2_O (Supplementary Fig. 5b), and the concentration was measured using the ultraviolet–visible light spectrophotometer.

### Enzymatic reactions

Typically, enzymatic reactions were performed with 10 µg (Figs. 1b, c, 2b, c, f, 4b, c and 5b, c and Supplementary Figs. 1, 2b,c,d,f, 4a and 5b,d,e) or 12 µg (Supplementary Fig. 2e) of labeled RNA at 37 °C. To digest RNA, samples were treated with 0.5 µl of RNase A (DNase and protease-free, 10 mg/ml) (Thermo Fisher Scientific, Figs. 1b, c, 2c, e, f, 4b, c and 5b, c and Supplementary Fig. 1, 2f, 4a and 5b,d), PureLink RNase A (20 mg/ml) (Thermo Fisher Scientific, Supplementary Fig. 5b) or 1 µL of RNase T1 (Thermo Fisher Scientific, Fig. 2f and Supplementary Fig. 2f) or 1 µl of RNase cocktail (500 U of RNase A and 20,000 U of RNase T1 per milliliter) (Thermo Fisher Scientific, Fig. 2f and Supplementary Fig. 2f) prepared in 1.5 µl of 10× TURBO DNase buffer (Thermo Fisher Scientific adjusting the final volume with Milli-Q H_2_O to 15 µl). To degrade DNA, samples were treated with 0.5 µl of TURBO DNase (2000 U/ml) (Thermo Fisher Scientific, Figs. 1b, c and 2c, e, f and Supplementary Figs. 1 and 2c,d,f) or DNase I (2,000 U/ml) (Thermo Fisher Scientific, Supplementary Fig. 2e) prepared in 1.5 µL of 10× DNase buffer adjusting the final volume with Milli-Q H_2_O to 15 µl. To digest both RNA and DNA, the samples were treated with 1 µl of benzonase (250,000 U/ml) (Merck, Fig. 2f and Supplementary Fig. 2f) or 1 µl of nuclease P1 (100,000 U/ml) (New England Biolabs, Fig. 2f and Supplementary Fig. 2f) prepared in 1.5 µl of 10× TURBO DNase buffer or nuclease P1 buffer adjusting the final volume with Milli-Q H_2_O to 15 µl. To digest RNA in DNA/RNA hybrids, samples were treated with 1 µl of RNase H (5000 U/ml) (New England Biolabs, Fig. 2f and Supplementary Fig. 2f) prepared in 1.5 µl of 10× RNase H buffer adjusting the final volume with Milli-Q H_2_O to 15 µl. To digest N-glycans, 0.5 µl of Rapid PNGase F (New England Biolabs, Fig. 2f and Supplementary Fig. 2f) were added with 1.5 µl of 10× PNGase F buffer (New England Biolabs) in the sample by adjusting with Milli-Q H_2_O to total 15 µl. To cut sialic acid moieties, 0.5 µl of α2-3,6,8,9 neuraminidase A (New England Biolabs, Fig. 2f and Supplementary Fig. 2f) were added with 1.5 µl of 10× GlycoBuffer 1 (New England Biolabs) in the sample by adjusting with Milli-Q H_2_O to total 15 µl. To digest proteins, 1 µl of proteinase K (Roche, 20 mg/ml dissolved in Milli-Q H_2_O, Figs. 1c, 2c, e, f, 4b, c and 5b, c and Supplementary Fig. 1, 2c,d,f, 4a and 5d, e; Thermo Fischer Scientific, 20 mg ml, Supplementary Fig. 2e) was added either with 1.5 µl of 10× TURBO DNase buffer in the RNA sample by adjusting with Milli-Q H_2_O to total 15 µl or directly in the precedent enzymatic reactant. The incubation was done for 30 or 60 min in cases, but all the results always exhibited complete protein digestion.

### RNA fragmentation

DBCO-Cy5-labeled RNA was fragmented using Ambion 10X RNA Fragmentation Reagent (Thermo Fisher Scientific). The samples were incubated in 1× RNA Fragmentation Reagent at 75 °C for 15 min for mild reaction (Supplementary Fig. 5d) and at 95 °C for 2 h for complete fragmentation (Supplementary Fig. 5e). Fragmented RNA samples were immediately mixed with 2× volumes of RNA binding buffer (RBB; RNA Clean and Concentrator-5 kit, Zymo Research) and various volumes of 100% ethanol for each sample for the final 40%, 50%, 60% and 70% ethanol, as indicated in Supplementary Fig. 5d,e.

### RNA clean-up by acidic phenol-chloroform extraction

For experiments in Figs. 1b and 2e, f and Supplementary Fig. 2d,e,f, enzymatically digested samples were mixed thoroughly with 1 ml of TRIzol reagent and 200 µl of 100% chloroform (Sigma-Aldrich) for 10 min at room temperature. The homogenates were centrifuged at 16,000*g* for 10 min at 4 °C. The upper phase was carefully removed, transferred into a nuclease-free sterile 1.7-ml tube and then mixed with 1 µl of linear acrylamide (Thermo Fisher Scientific) as a coprecipitant and equal volume of 100% isopropanol by vortexing, subsequently centrifuged at 16,000*g* for 30 min at 4 °C, and the supernatant was carefully discarded. The pellet was washed with 1 ml of ice-cold 75% ethanol twice and then dried completely. The pellet was dissolved with Milli-Q H_2_O. For experiments in Supplementary Fig. 2e, 20 µg linear acrylamide and TRI Reagent Solution (Thermo Fisher Scientific) were used.

### RNA clean-up and size fractionation by silica-based column purification

A total of 16 µl of proteinase K-digested samples were mixed with 34 µl of Milli-Q H_2_O. A total of 100 µl of RBB and 150 µl of 100% ethanol (final 50% ethanol) were added by reverse pipetting and vortexed thoroughly. For experiments shown in Supplementary Fig. 2e, the final percentage of EtOH was 60%. The mixtures were transferred into the Zymo-SpinTM IC column in a 2 ml collection tube. The columns were centrifuged at 16,000*g* for 30 s at room temperature, and the flow-through was discarded. A total of 400 µl of RNA prep buffer (RPB; RNA Clean and Concentrator-5 kit, Zymo Research) were added into the column and then centrifuged at 16,000*g* for 30 s at room temperature followed by discarding the flow-through. A total of 700 µl of RNA wash buffer (RWB; RNA Clean and Concentrator-5 kit, Zymo Research) were added into the column and then centrifuged at 16,000*g* for 30 s at room temperature, followed by discarding the flow-through. A total of 400 µl of RWB were added into the column and then centrifuged at 16,000*g* for 30 s at room temperature, followed by discarding the flow-through. To ensure complete removal of the RWB, samples were again centrifuged at 16,000*g* for 1 min at room temperature. The columns were carefully transferred into a new nuclease-free sterile 1.7-ml tube, and 15 µl of Milli-Q H_2_O or DEPC-treated H_2_O was directly applied to the column matrix and incubated for 3 min. The elution was done by centrifugation at 16,000*g* for 3 min at room temperature.

For size fractionation of small (smaller than about 200 nucleotides) versus large (larger than about 200 nucleotides) RNAs, samples were mixed with equal volume of 50% RBB in 100% ethanol. The mixture was applied onto the Zymo-SpinTM IC Column and centrifuged at 16,000*g* for 1 min at room temperature. Large RNAs bound in the column were purified as described above. Small RNAs in the flow-through were mixed with equal volume of 100% ethanol and then purified as described above.

### Photochemical labeling of fragmented RNA with amine-DBCO and click chemistry

The purified RNA sample was fragmented using the NEBNext Magnesium RNA Fragmentation Module (E6150S) following protocols recommended by the vendor and purified using Zymo RCC-25. Amine-DBCO (CAS no. 1255942-06-3) methylene blue (CAS no. 61-73-4) was added to a solution of 50 µg RNA at a final concentration of 1 mM and 100 µM, respectively. The solution was exposed to red light (50 W light-emitting diode chip on board) on ice for 15 min with lid open. The resulted reaction mixture was then purified using TRIzol in combination with Zymo RCC-25 following the vendor-provided protocol. The purified DBCO-functionalized RNA (10 µg per reaction) was then clicked with either Cy5-azide (Jena Bioscience, CLK-1177) or the AlexaFluor-555-labeled sialoglycan-azide at 200 and 60 µM, respectively, at 50 °C for 10 min. The clicked RNA product was then purified with TRIzol in combination with Zymo RCC-10 following the vendor’s protocol. The RNA samples were resolved with 1% denaturing agarose gel and fluorescently imaged under a GelDoc scanner, after which the gel was stained with ethidium bromide (EtBr) and scanned again for total RNA.

To make AlexaFluor-555-labeled sialoglycan-azide, G2 glycoform N-glycan linked with an azido-asparagine (final concentration 120 µM) was mixed with 10 µg ST6Gal1 and 200 µM cytidine monophosphate–sialic acid prefunctionalized with AlexaFluor-555. The sialylation reaction was incubated at 37 °C for 48 h and was used for the click chemistry with RNA directly without further purification.

### Denaturing gel electrophoresis, membrane transfer and blotting

Typically, formaldehyde-denaturing 1% agarose gels were prepared by thoroughly mixing 0.5 g of agarose powder (Lonza) with 45 ml of Milli-Q H_2_O in an Erlenmeyer flask. The agarose was melted by heating in the microwave oven and then cooled to 55–60 °C. A total of 5 ml of 10× NorthernMax denaturing gel buffer (Thermo Fisher Scientific) were added and mixed by swirling in the fume hood. The gel mixture was casted following the instruction provided by the casting apparatus. To prepare loading samples, the samples were mixed with equal volume of 2× dye-free RNA loading buffer and then incubated at 95 °C for 10 min. The gel was resolved in 1× NorthernMax running buffer (Thermo Fisher Scientific) at 100 V for 40–50 min. For visualization of the Cy5 fluorescence, the gel was rinsed briefly with Milli-Q H_2_O and scanned using the gel imaging system (Bio-Rad ChemiDoc XRS+) in the Cy5 filter channel. For EtBr scanning, the Cy5 scanned gel was stained in the water-dissolved UltraPure EtBr (Thermo Fisher Scientific) solution for 30 min at room temperature by rocking. The gel was rinsed with Milli-Q H_2_O for 30 min at room temperature by rocking and then scanned in the gel imaging system.

For experiments shown in Supplementary Fig. 2e, 1 g of agarose powder (Roche) was dissolved in 72 ml of Milli-Q H_2_O. A total of 10 ml of 10× 3-(*N*-morpholino)propanesulfonic acid (MOPS) buffer (200 mM MOPS, 50 mM sodium acetate·3H_2_O, 10 mM ethylenediaminetetraacetic acid, pH 7.0) and 18 ml 37% formaldehyde (Merck) were added and mixed. Purified, enzyme treated, RNA samples were incubated at 95 °C for 5 min, followed by a quick transfer and 5 min incubation on ice before gel electrophoresis at 90 V. Cy5 fluorescence was visualized using the Amersham Typhoon scanner (GE Healthcare).

For membrane transfer, the electrophoresed gel was scanned in the Cy5 channel and then immediately subjected to the transfer instead of EtBr staining, as the EtBr emission was strongly overlapped with Cy5 visualization during the downstream membrane scanning. NorthernMax transfer buffer (Thermo Fisher Scientific) was used following the manufacturer’s instruction for 2 h. For nylon membranes, Zeta-Probe GT (Bio-Rad) or BrightStar-Plus (Thermo Fisher Scientific) membranes were used. For nitrocellulose membranes, Hybond-C (Cytiva) or Amersham Protran (Sigma-Aldrich) membranes were used. The transferred membranes were rinsed briefly with Milli-Q H_2_O and scanned immediately in the Cy5 channel using Bio-Rad ChemiDoc XRS+.

After transfer of the gel run with biotinylated samples, the membranes were subjected to blocked with Odyssey blocking buffer, PBS (Li-Cor Biosciences) for 45 min at room temperature, by skipping EtBr staining and fluorescent imaging. After blocking, the membranes were stained for 30 min at room temperature with streptavidin-conjugated IR800 (Li-Cor Biosciences), which was diluted to 1:5,000 in Odyssey blocking buffer. Excess IR800-streptavidin was removed from the membranes by four washes with 0.1% Tween-20 (Sigma-Aldrich) in 1× PBS for 10 min each at room temperature. The membranes were finally washed once with 1× PBS to remove residual Tween-20 before scanning. Fluorescent signals from membranes were scanned on Odyssey Li-Cor Sa scanner (Li-Cor Biosciences) with the software set to autodetect the signal intensity for both 700 and 800 channels. After scanning, the images were adjusted to appropriate contrasts with the Li-Cor software (when appropriate) in the 800 channel and exported.

## Results

### Recapitulation of reported observation about glycoRNA

The originally reported workflow for glycoRNA isolation starts by supplementing the cell culture with Ac_4_ManNAz, which is biosynthetically converted to SiaNAz on the glycans. This is followed by extracting the total RNA using TRIzol and cleaning up RNA samples using multiple enzymes including proteinase K and a deoxyribonuclease (DNase). More digestive enzymatic treatments are performed to provide indications of the chemical nature of the isolated molecules. The treatment includes ribonucleases (RNases) or glycan hydrolases, either of which is expected to result in a loss of glycoRNA. As the final step of sample treatment, a DBCO-biotin is clicked onto the sialic acid residue to allow biochemical detection on a biotin blot. Because the DBCO-biotin click chemistry is performed as the last step after all digestive enzymatic treatment, we refer to this reported workflow as the ‘late-click procedure’. It is important to highlight that in between each sample treatment, there is an RNA purification step using a commercial microscale silica column. Finally, upon visualization, glycoRNA signal is considered to have a high apparent molecular weight (MW), which is around that of the 28S ribosomal RNA (rRNA) band.

We first sought to reproduce the reported observation. Following the reported workflow, especially the silica column purification in between steps and a late-click procedure (Fig. 1a, left), we recapitulated the reported observation (Fig. 1b, left), in which a band was shown close to the 28S rRNA MW, albeit we clicked with DBCO-sulfoCy5 and employed in-gel fluorescence for visualization. As reported, when the sample was treated with RNase A before the click reaction, the fluorescent band with high apparent MW was lost. To ensure in-gel fluorescence does not introduce unwanted artefacts, we also performed biotin blot on either nitrocellulose (NC) or nylon membranes for comparison and observed a similar MW of the biotin signal as sulfoCy5-clicked sample, as well as its sensitivity for RNase treatment (Supplementary Fig. 1). These results demonstrate the previous observation can be reproduced not only when the reported procedure is followed but also when adaptive changes, such as the visualization method, are introduced. Because in-gel fluorescence eliminates the need for membrane transfer, we employed this method hereafter due to its practicality.

Because ManNAz-based metabolic labeling is not specific to glycoRNA and also labels other glycoconjugates, which might contaminate RNA sample preparation, the click reaction was deliberately moved to an earlier stage before the digestive enzymatic treatment (Fig. 1a, right). The modified workflow (referred to as the ‘early click procedure’) would allow us to see if any glycosylated, non-RNA species could be copurified. Following the early click procedure, with silica column purification after the digestive enzymatic treatment, we found that the modified workflow also recapitulated the previous observation: the same high-MW band, close to the 28S rRNA position, was detectable (Fig. 1b, right) and was lost after RNase treatment. Interestingly, when proteinase K treatment was skipped, we did observe an additional high-MW band right below the presumable glycoRNA band, probably given rise to by copurified glycopeptides (Fig. 1c). Taken together, the results suggest while our early and the reported late-click procedures both work to afford the same product, the former allows visualization of more species such as glycopeptides, which may be carried over along the sample preparation.

### RNA purification methods cause discrepancies in RNase sensitivity

We sought to further examine the specificity of glycoRNA detection. We reasoned that a post-transcriptionally modified RNA should exhibit general properties of RNAs and should be isolated with orthogonal RNA-focused purification techniques other than silica columns. We therefore designed workflows that bypass silica column purification. In the independent workflow, we have minimized the RNA purification steps: the post-click RNA sample was first subjected to nuclease digestion, and then the mixture was directly subjected to proteinase K digestion in the same tube and then loaded into agarose gel without further RNA purification (Fig. 2a). No fluorescent signal was observed in RNA obtained from DMSO-treated negative control cells (Fig. 2b). For ManNAz-treated cells, we observed a high-MW band at the same position as in previous experiments. This high-MW band was also more enriched in small RNA fraction (<200 nt) when using silica column to fractionate the extracted total RNA (Fig. 2c). This was in line with the previous reports on glycoRNA, in which the signal coincided with small RNA. However, in stark contrast to silica column purification, the SiaNAz-labeled fluorescent band remained as strong even after an extended RNase treatment in the silica column-independent methods, whereas total RNA was completely degraded (Fig. 2c).

The persistence of the SiaNAz-containing band after RNase digestion was not related to visualization methods, as we observed the RNase resistance in all methods employed (in-gel fluorescence and RNA blotting to nitrocellulose or nylon membranes) (Supplementary Fig. 2a–c). Thus, we argue that the key difference which has led to the discrepancies in the RNase sensitivity is in the RNA purification methods after the RNase treatment. The observation promoted us to hypothesize that the silica column purification following the RNase digestion step had been the cause for the apparent RNase sensitivity. To test this hypothesis, we designed a pair of comparable workflows (Fig. 2d). All other sample processing steps were kept identical, except for the RNA purification step following RNase digestion. The digested sample was either purified with the silica column or AGPC (TRIzol) with a subsequent ethanol precipitation. Consistent with the results above, we found that the SiaNAz-containing molecules exhibit the same MW regardless of purification methods (Fig. 2e), yet the recovery of the SiaNAz-modified molecules was substantially poorer when silica columns were used to clean up the sample. Importantly, while the RNase activity was confirmed by the degradation of total RNA (EtBr channel), the loss of fluorescent glycan signal upon RNase-treatment was only observed when silica column-based purification was used (Fig. 2e and Supplementary Fig. 2d,e) but not for the TRIzol method. In previous reports, the loss of SiaNAz signal upon RNase digestion has been used as a key indicator for a successful isolation of glycoRNA^1^. Here, we show the routinely used silica column in glycoRNA purification can cause an apparent RNase sensitivity for an RNase-resistant sialoglycoconjugate. Such RNase sensitivity became absent when an orthogonal RNA purification method was employed.

### The RNase-resistant glycoconjugate is modified by N-glycan but is not an RNA molecule

To get an indication for the chemical nature of the RNase-resistant species, we further investigated the reactivity of the RNase-resistant glycoconjugate toward a wider array of enzymes. We observe that, regardless of whether RNA was purified with silica columns or TRIzol extraction followed by ethanol precipitation, the glycan signal was sensitive to the treatment with PNGase F and α2-3,6,8,9 neuraminidase A (Fig. 2e and Supplementary Fig. 2d,e). The former is an amidase that cleaves oligomannose-, hybrid- and complex-type N-glycans from glycoproteins/peptides^16^, and the latter is an exoglycosidase that removes terminal sialic acid residues linked to a penultimate sugar^17^. Our results suggest that the detected glycosylated molecules in our experiments contain hybrid or complex N-glycans and exhibit the highly similar reactivities toward the digesting enzymes as reported for glycoRNA^1^. The assays described above have largely relied on RNase A and/or T1. To exclude that RNase resistance of the N-glycoconjugate had been due to the enzyme’s substrate specificity, we performed digestion assays on the glycan-labeled molecule with an expanded collection of nucleases. While RNase A catalyzes the cleavage of single-stranded RNA after pyrimidine nucleotides^18^, RNase T1 specifically degrades single-stranded RNA at G residues^19^; benzonase can degrade various forms of DNA and RNA^20^; RNase H cleaves RNA in RNA:DNA hybrids^21^; and nuclease P1 hydrolyzes phosphodiester bonds in RNA and single-stranded DNA without base specificity^22^. Our results demonstrated that RNase cocktail, comprising RNase A and T1, and benzonase degraded RNA completely, and RNase T1 alone and nuclease P1 digested almost all RNA into small pieces, whereas RNase H did not result in RNA degradation (Fig. 2f and Supplementary Fig. 2f). Under all these conditions, signals corresponding to the N-glycoconjugate remained after TRIzol purification and the ensuing ethanol precipitation clean-up. The resistance of the N-glycoconjugate toward multiple nucleases supports it is not a nucleic acid. By contrast, with silica column as the final clean-up step, the N-glycoconjugate signal disappeared in all samples with degraded total RNA (Fig. 2f and Supplementary Fig. 2f). Taken together, the association with the intact RNA appears to be crucial for the recovery of the N-glycoconjugate over a silica column, but the presence of RNA is not required when the same molecule is precipitated after TRIzol purification.

**Fig. 3:**
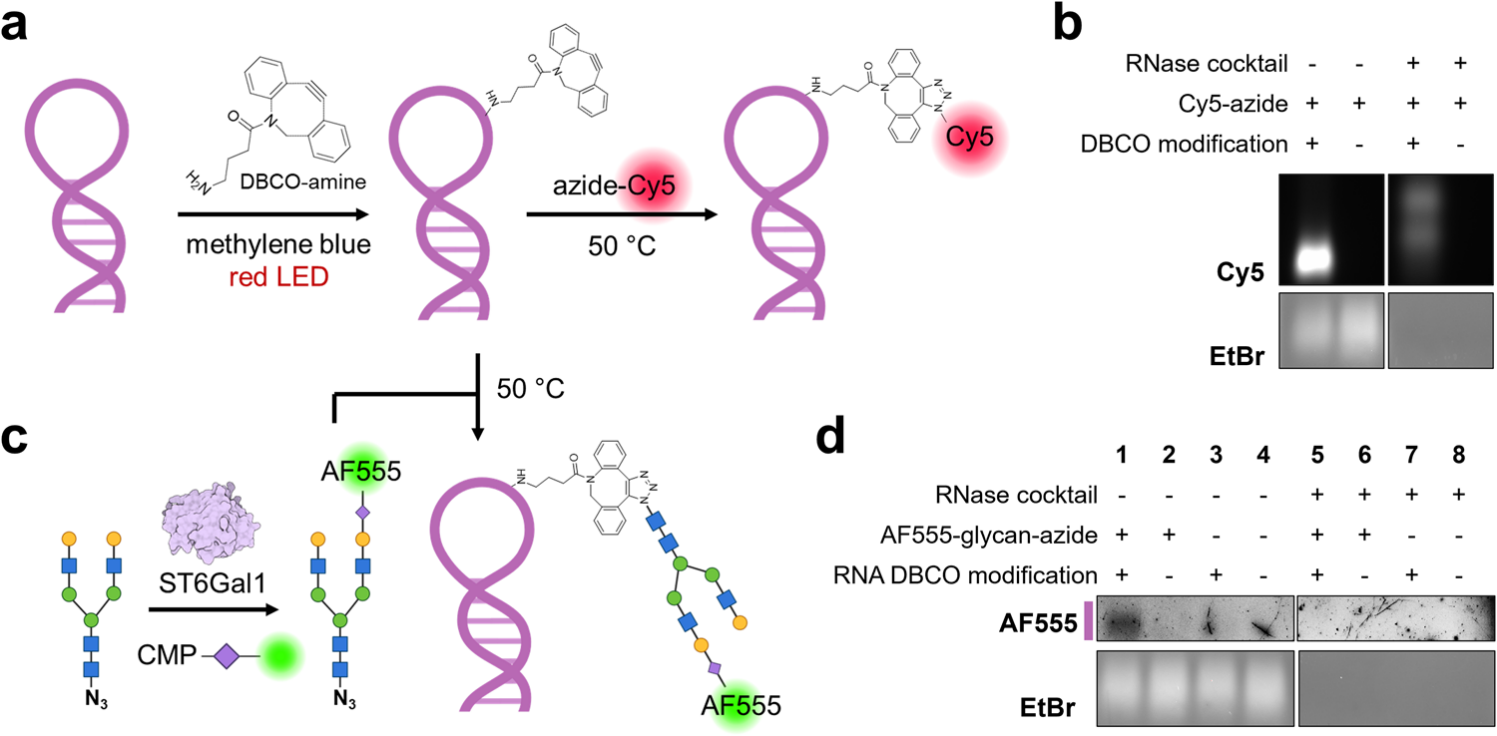
Chemically prepared neo-glycoRNA has different properties from N-glycoconjugates. **a** Schematics for preparing DBCO-functionalized RNA for click strain-promoted click reaction. **b** Denaturing agarose gel (1%) electrophoresis and in-gel fluorescence of Cy5-clicked, DBCO-functionalized RNA. The EtBr-stained gel was scanned after the fluorescence scan of the same gel. **c** Schematics for preparing N-glycan conjugated neo-glycoRNA. **d** Denaturing agarose gel (1%) electrophoresis and in-gel fluorescence of neo-glycoRNA and RNase-treated samples. The pink line next to the AF555 fluorescent gel indicates the position of the EtBr signal of the same gel. The AF555 signal has a highly similar mobility to EtBr signals. AF555, AlexaFluor-555; CMP, cytidine monophosphate; DBCO, dibenzocyclooctyne; LED, light-emitting diode; ST6Gal1, β-galactoside α-2,6-sialyltransferase 1.

Next, we took a synthetic chemical approach to assess the chemical properties of artificially glycosylated RNA molecules to demonstrate the N-glycoconjugate is unlikely RNA. We asked how RNA would migrate in agarose gels if it were covalently attached to an N-glycan and whether the glycan can confer resistance toward RNase. However, it has not been possible to build glycoRNA in vitro, although a recent publication has provided structural indications for the chemical linkage between N-glycans and RNA^23^. We therefore sought to make an artificially N-glycosylated RNA (neo-glycoRNA) to test its enzymatic reactivities and electrophoretic properties. Extracted total cellular RNA was first fragmented into small fragments by magnesium. We then introduced a strained alkyne (DBCO) to the guanosine residues via a photochemical reaction (Fig. 3a). Upon irradiation by red light, methylene blue generates singlet oxygen, turning guanine into a reactive intermediate, which reacts with a primary amine functionalized DBCO^24^. A biantennary galactose-terminated N-glycan with asparagine at its reducing end was obtained from a sialoglycopeptide extracted from egg yolk^25^ (see Supplementary Fig. 3a for a proton nuclear magnetic resonance spectrum). The α-amino group was then converted to an azide, which permits later strain-promoted click reaction with DBCO. The azide-containing N-glycan was first sialylated with an AlexaFluor-555-modified sialic acid derivative by human sialyltransferase ST6Gal-I^26^ and then directly mixed with DBCO-modified RNA fragments to afford the neo-glycoRNA (Fig. 3c). We expect such entity to be structurally analogous to glycoRNA (Supplementary Fig. 3b), especially as guanosine had been proposed as an N-glycan attachment site^27^, although the modified uridine (acp^3^U) was experimentally supported to carry N-glycans^23^.

To confirm the photochemical incorporation of DBCO, we included a positive control, in which an azide-functionalized fluorophore (Cy5) was clicked with DBCO-conjugated RNA (Fig. 3a). The chemically modified RNA samples were subjected to agarose gel electrophoresis after the purification. The strong Cy5 signal demonstrated the successful incorporation of DBCO into the RNA (Fig. 3b). Little, if any, nonspecific interaction between the dye and the RNA was observed, as no fluorescence was observed for unmodified RNA. Similarly, when DBCO-RNA was clicked to the fluorescently labeled N-glycan, a fluorescent band was also observed. Interestingly, in sharp contrast to the previously observed N-glycoconjugates, the neo-glycoRNA had a similar apparent MW in the agarose gel as its unmodified counterparts (Fig. 3d, lane 1), as evident by similar migration behavior of the EtBr-stained bands. Thus, whereas Fig. 2e demonstrates that the copurified N-glycoconjugate can migrate at similar MWs to the putative glycoRNA signal, incorporation of authentic glycans into small RNAs does not appear to substantially alter their migration in gels. Furthermore, the fluorescent band was lost upon RNase treatment right before sample loading into gels (lane 5), suggesting N-glycans do not confer any RNase resistance to modified RNA, further suggesting that the N-glycoconjugate is not RNA and that the previously observed mobility shift must be explained by other structural features^28^.

### Increased alcohol concentrations enhance the silica adsorption of RNA-associated N-glycoconjugate

**Fig. 4:**
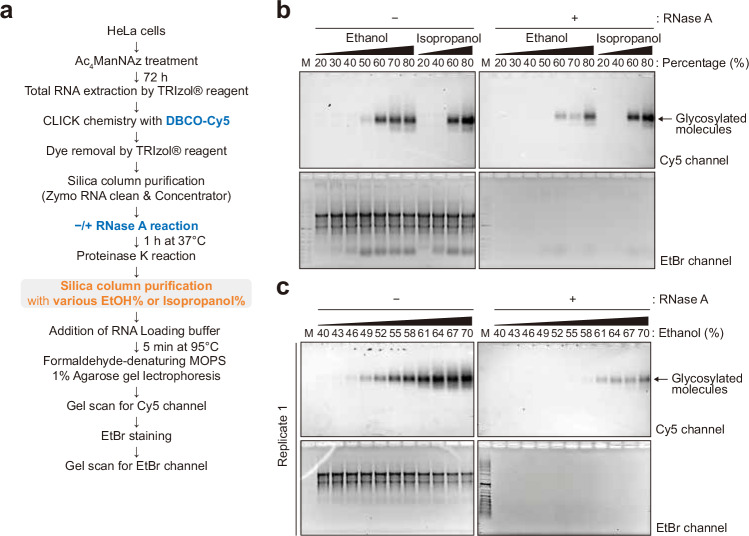
Ethanol percentage and RNA existence are critical for glycosylated molecule binding onto silica column. **a** A schematic of glycan visualization protocol for various ethanol or isopropanol percentage in silica column binding solutions. **b** Glycan detection in 20–80% ranges of ethanol or isopropanol by 10% increments. **c** Glycan detection in 40–70% ranges of ethanol by 3% increments.

We next sought to understand why the N-glycoconjugate appeared to be sensitive to RNase only when silica columns are used for its capture and elution. Silica-based solid-phase capture of RNA typically uses a solution of 50% ethanol to promote the capture of biomolecules on the column, which after washing is then eluted using a more polar 100% aqueous solution. We hypothesized that for a polar N-glycoconjugate (for example, an N-glycan-modified peptide), its capture might be sensitive to the presence of RNA in solution, whose aromatic nucleobases are nonpolar and could contribute to column capture. To test this hypothesis, we first simply varied the ethanol or isopropanol percentage from 20% to 80% in the sample loading buffer when purifying RNase-treated and untreated samples using the silica column, predicting that a higher percentage of ethanol would promote increased capture (Fig. 4a). We found that the N-glycoconjugate was not captured at ethanol concentrations below 40%, regardless of RNase treatment. In line with our previous observations, at 50% ethanol concentration, the glycoconjugate was recovered in untreated conditions but not in the RNase-treated condition (Fig. 4b, c and Supplementary Fig. 4). When the percentage of ethanol increased, the glycoconjugate was recovered more efficiently (Fig. 4b, c and Supplementary Fig. 4). At around 60% ethanol concentrations and above, the glycan signal became visible even in the RNase-treated condition (Fig. 4b, c and Supplementary Fig. 4). Similar results were obtained when ethanol was replaced with isopropanol (Fig. 4b). Our results on the correlation between the polarity of the loading buffer and the recovery efficiency of the N-glycoconjugate pointed to a co-adsorption or coprecipitant effect of RNA on the former and further strengthened our claims that the N-glycoconjugate had been physically depleted over the silica column in the absence of RNA but not digested by RNase.

### Exogenous RNA facilitates the adsorption of the N-glycoconjugate on silica columns

**Fig. 5:**
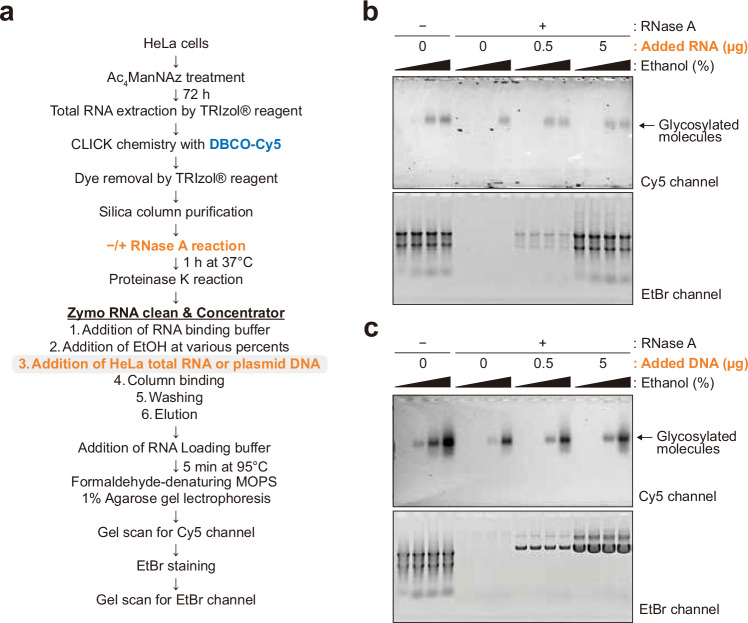
RNA is the cobinder of glycosylated molecules during silica column purification. **a** A schematic of glycan visualization protocol for RNA or DNA addition in silica column binding solutions. A total of 5 µg of total RNA extracted from Ac_4_ManNAz-treated HeLa cells were subjected to click chemistry per sample. **b** Glycan detection without or with total RNA addition in RNA-depleted samples. The amounts of added total RNAs extracted from DMSO-treated HeLa cells are indicated. The range of the ethanol concentrations is 40–70%. **c** Glycan detection without or with plasmid DNA addition in RNA-depleted samples. The amounts of added plasmid DNA are indicated. The range of the ethanol concentration is 40–70%.

Our observation indicates that the N-glycoconjugate in RNA preparations are not digested by RNase but did become less adsorbed on silica columns at around 50% ethanol concentration when RNA had been degraded. This led us to hypothesize that the total RNA, regardless of sources, has an enhancing effect on the silica adsorption of the N-glycoconjugate. On this basis, we should expect a heightened recovery of the N-glycoconjugate over silica column when exogenous RNA is added back in the loading buffer. We thus performed a rescue experiment by adding total RNA extracted from unlabeled HeLa cells (that is, not exposed to Ac_4_ManNAz) to the RNase-treated sample (Fig. 5a). To ensure newly added RNA was not degraded by residual RNase activity, we removed RNase thoroughly by treating the samples with proteinase K followed by TRIzol extraction before the addition of new total RNAs. Strikingly, exogenously added, unlabeled total RNA effectively reduced the required minimum ethanol percentage for binding of the N-glycoconjugate to silica columns (Fig. 5b). Adding only one tenth of the amount of RNA typically present in our RNA preparation was sufficient to fully restore recovery (Fig. 5b). Similarly, unlabeled RNA from a different cell line (K562) also enhanced binding efficiency of the N-glycoconjugate (Supplementary Fig. 5a,b).

Moreover, partially fragmented RNA was as sufficient in coprecipitating the N-glycoconjugate (Supplementary Fig. 5c,d). However, more intense RNA fragmentation, which resulted in almost complete RNA degradation, did not have the enhancing effect on N-glycoconjugate recovery, an observation akin to RNase treatment. Adding exogenous total RNA to the samples depleted for RNA by nonenzymatic fragmentation also rescued the binding of the N-glycoconjugate to silica matrix (Supplementary Fig. 5e). Interestingly, the addition of plasmid DNA had little effect on the minimum ethanol percentage required for binding of the N-glycoconjugate to silica (Fig. 5c). These results suggest RNA—but not double-stranded DNA—may interact with the N-glycoconjugate, leading to their efficient co-isolation in silica-column-based extraction methods. Our rescue experiment also demonstrates that the N-glycoconjugate is unlikely covalently attached to RNA, despite its persistence throughout RNA preparations.

## Discussion

The recent description of N-glycosylated small RNA molecules has implications for RNA biology, the cellular trafficking of RNA and the substrate specificity of glycosylating enzymes, as well as expanded the repertoire of post-transcriptional RNA modifications^1^. Despite at its infancy, the glycoRNA field has recently seen considerable developments regarding its biological functions in cancer and innate immunity^4,5^, as well as the indication of its chemical nature^23^. In all studies, biochemically characterizing glycoRNA using electrophoresis has been essential for supporting the conclusions in the published work. Our findings provide critical methodological considerations for this nascent field, describing that a non-RNA N-glycoconjugate as an independent, separate molecular entity with broadly similar properties to glycoRNA may confound the biochemical analysis of the latter. It is also crucial to note that in our work, we initiated our investigation employing a procedure independent of the one in prior publications, attempting to maximize the recovery of glycoRNA and presuming that independent procedures should serve to strengthen a biological observation. We compared our procedure with the reported workflow (referred to as ‘late-click’ procedure in this manuscript). Both methods initially gave consistent observations, yet the higher yield of total RNA together with the possibility to control for additional associated molecular entities justified following up on our own ‘early click’ procedure.

The non-RNA N-glycoconjugates persist in RNA preparations from mammalian cells even after multiple clean-ups and are resistant to digestion by multiple enzymes, including RNases, DNases and proteinase K. The N-glycoconjugates also remain with RNA during phenol-chloroform phase separation and ethanol precipitation-based purification. The non-RNA nature of the N-glycoconjugates was further indicated by their drastic difference from the chemically prepared neo-glycoRNA, in which the attachment of glycans did not confer RNA the protection from degrading enzymes. However, these persisting N-glycoconjugates did become depleted, but not digested, only when using silica columns to purify the RNase-treated samples. Given that assays on glycoRNA also routinely employ silica columns for the final clean-up before electrophoresis, it is thus difficult to distinguish the two scenarios when the loss of the glycan-labeled bands is observed: Should the loss of signals be interpreted as (1) RNase digestion of glycoRNA or (2) the physical depletion of N-glycoconjugates upon RNA removal?

To address this question, we propose a simple checkpoint experiment for the relevant fields, as was performed in our study. This approach can help distinguish whether the detected molecule is true glycoRNA or a non-RNA N-glycoconjugate. We suggest treating the purified, glycan-labeled RNA samples with RNases for an extended period and then directly load the mixture into the gel for electrophoresis, without using a column to clean up the sample, regardless of whether the sample preparation follows an early click or a late-click procedure as reported. With a transfer to membrane or not, the band at large MWs should disappear for glycoRNA. If it does not, one should be alerted that the non-RNA N-glycoconjugates have been largely co-isolated.

Our observation prompted us to investigate the mechanism of the association of the N-glycoconjugates during silica column purification. First, with varying ethanol concentration or switching to isopropanol during sample loading, we observed that the N-glycoconjugate adsorption follow the general rules for silica-based systems: the more apolar content (that is, ethanol or isopropanol) in the loading buffer, the better the molecules are retained on the silica resin. Although 60% or higher ethanol or isopropanol concentrations in the loading buffer deviates from the vendor-suggested alcohol content for RNA sample loading, it did substantially improve the recovery of the N-glycoconjugates over a silica column, especially compared with that when RNA had been removed before sample loading. Notably, glycoRNA has been shown to exhibit similar adsorption properties on silica^23^. Secondly, the N-glycoconjugate is not covalently attached to RNA. However, exogenously added RNA regardless of sources of extraction can enhance the adsorption of the N-glycoconjugates to silica resin, indicating that RNA acts as a coprecipitant for the N-glycoconjugates.

While the chemical nature of the newly characterized N-glycoconjugates in this work remain to be elucidated, we propose N-glycosylated peptides as a candidate which warrant follow-up confirmative studies. N-glycans themselves may contain negatively charged moieties, such as sialic acid, phosphate or sulfate groups^29–34^, which may contribute to their mobility toward the positive electrode during gel electrophoresis and efficient transfer to a positively charged nylon membrane. Thus, potential candidates of these molecules may be N-glycosylated oligopeptide products degraded from glycoproteins, which cannot be further cleaved by proteinase K. The possible peptidic nature of glycoRNA-associated glycosylated molecules is supported by the cleavage of an N-glycan from asparagine residues by PNGase F, which requires at least a tripeptide-containing substrate^35^. Interestingly, a highly hydrophilic oligopeptide containing a sialylated complex-type N-glycan linked to a hexapeptide can be isolated from chicken egg yolk in considerable quantity and high homogeneity^25^. It is thus intriguing to ask if similar molecules also exist in mammalian cells.

Of general importance, our findings demonstrate that even the gold standard RNA purification methods are susceptible to seemingly inert molecules, such as glycans, which are not easily detected by conventional means. It should be brought to the attention that co-isolation of other biomolecules with extracted nucleic acids are not uncommon. For example, anticoagulant heparin often contaminates purified DNA or RNA samples from blood collection and plasma processing procedures, and such contamination can complicate reverse transcription and PCR analysis^14,15^. It is currently unclear how glycosylated molecules may have affected and will affect studies that have relied on conventional RNA isolation methods. Our work prompts the development of more reliable RNA purification and post-transcriptional modification methods and will serve as a catalyst for further investigation into a potentially novel biomolecule.

## Supplementary information

Supplementary Information

## Data Availability

All unique/stable reagents generated in this study are available from the lead contact with a completed Materials Transfer Agreement.
