# Supplementary material for: RNA-associated glycoconjugates highlight potential ambiguities in glycoRNA analysis
Source: Exp Mol Med. 2025 Nov 15;57(11):2619–28. doi: 10.1038/s12276-025-01585-z (PMC12685941; doi:10.1038/s12276-025-01585-z)
Supplement: Supplementary file 1 — Supplementary Information [file 12276_2025_1585_MOESM1_ESM.pdf]

Supplementary Information for

RNA-associated glycoconjugates highlight potential ambiguities in glycoRNA analysis

Sungchul Kim[1,2,3,12,§], Zeshi Li[4,12,§], Yong-geun Choi[1], Kirsten Janssen[5], Jan-Willem H. Langenbach[4], Daan J. van den Brink[4], Christian Büll[6], Bhagyashree S. Joshi[7,13], Adam Pomorski[7,8], Vered Raz[9], Marvin E. Tanenbaum[7,10], Pascal Miesen[5,§], and Chirlmin Joo[7,11,§]

[1] Center for RNA Research, Institute for Basic Science, Seoul 08826, Republic of Korea

[2] Department of Life Sciences, Pohang University of Science and Technology, Pohang, Gyeongbuk, 37673, Republic of Korea

[3] Institute of Convergence Science, Yonsei University, Seoul, 03722, Republic of Korea

[4] Division of Chemical Biology & Drug Discovery, Utrecht Institute for Pharmaceutical Sciences, Utrecht University, 3584 CG Utrecht, The Netherlands

[5] Department of Medical Microbiology, Radboud University Medical Center, 6525 GA Nijmegen, The Netherlands

[6] Department of Biomolecular Chemistry, Institute for Molecules and Materials, Radboud University, 6525 AJ, Nijmegen, The Netherlands

[7] Department of BioNanoScience, Kavli Institute of Nanoscience Delft, Delft University of Technology, 2629 HZ, Delft, The Netherlands

[8] Department of Chemical Biology, Faculty of Biotechnology, University of Wrocław, 50-383, Wrocław, Poland

[9] Human Genetics department, Leiden University Medical Centre, 2333ZC Leiden, The Netherlands

[10] Oncode Institute, Hubrecht Institute–KNAW and University Medical Center Utrecht, 3584 CT Utrecht, The Netherlands

[11] Department of Physics, Ewha Womans University, Seoul 03760, Republic of Korea

[12] These authors contributed equally.

[13] Present address: Whitehead Institute for Biomedical Research, Cambridge, MA 02142, U.S.A.

[§] Correspondence: sungchulkim.kr@gmail.com, pascal.miesen@radboudumc.nl, z.li@uu.nl, c.joo@tudelft.nl

Supplementary Figures

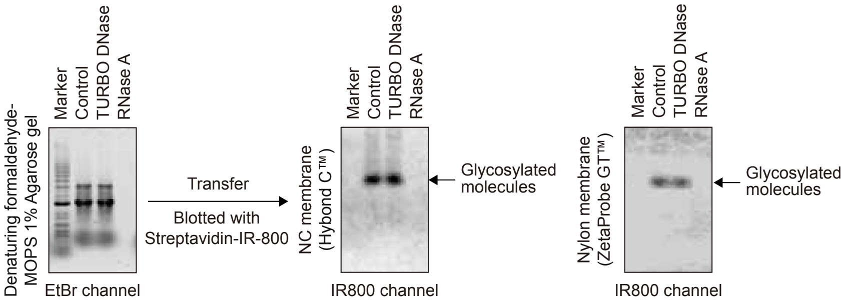

Supplementary Figure 1. Membrane transfer assay for blotting-based glycan detection.
Blotting of biotinylated glycans using streptavidin-conjugated IR800 dyes on the nitrocellulose and nylon membrane. Right, gel image of EtBr-stained RNA samples. Left, fluorescent image using IR800-streptavidin in 800-nm channel.

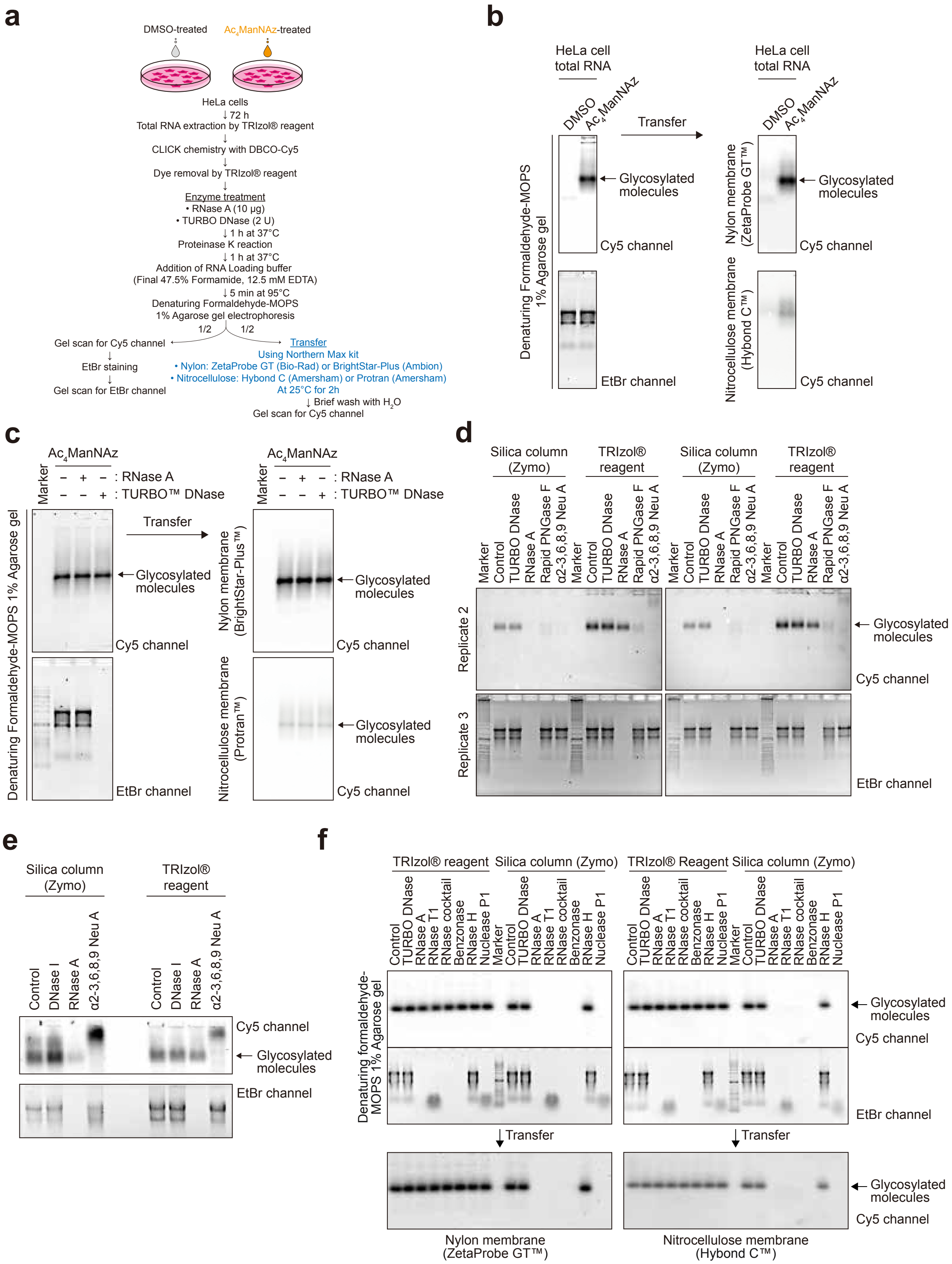
a
DMSO-treated
Ac4ManNAz-treated
HeLa cells
72 h
Total RNA extraction by TRIzol® reagent
CLICK chemistry with DBCO-Cy5
Dye removal by TRIzol® reagent
Enzyme treatment
• RNase A (10 μg)
• TURBO DNase (2 U)
1 h at 37°C
Proteinase K reaction
1 h at 37°C
Addition of RNA Loading buffer
(Final 47.5% Formamide, 12.5 mM EDTA)
5 min at 95°C
Denaturing Formaldehyde-MOPS
1% Agarose gel electrophoresis
1/2
1/2
Gel scan for Cy5 channel
EtBr staining
Gel scan for EtBr channel
Transfer
Using Northern Max kit
• Nylon: ZetaProbe GT (Bio-Rad) or BrightStar-Plus (Ambion)
• Nitrocellulose: Hybond C (Amersham) or Protran (Amersham)
At 25°C for 2h
Brief wash with H2O
Gel scan for Cy5 channel
b
HeLa cell total RNA
DMSO
Ac4ManNAz
Transfer
Glycosylated molecules
Cy5 channel
EtBr channel
Denaturing Formaldehyde-MOPS 1% Agarose gel
Nylon membrane (ZetaProbe GT™)
Nitrocellulose membrane (Hybond C™)
c
Ac4ManNAz
Marker
: RNase A
: TURBO™ DNase
Transfer
Glycosylated molecules
Cy5 channel
EtBr channel
Denaturing Formaldehyde-MOPS 1% Agarose gel
Nylon membrane (BrightStar-Plus™)
Nitrocellulose membrane (Protran™)
d
Silica column (Zymo)
TRIzol® reagent
Marker
Control
TURBO DNase
RNase A
Rapid PNGase F
α2-3,6,8,9 Neu A
Replicate 2
Replicate 3
Glycosylated molecules
Cy5 channel
EtBr channel
e
Silica column (Zymo)
TRIzol® reagent
Control
DNase I
RNase A
α2-3,6,8,9 Neu A
Cy5 channel
Glycosylated molecules
EtBr channel
f
TRIzol® reagent
Silica column (Zymo)
TRIzol® Reagent
Control
TURBO DNase
RNase A
RNase T1
RNase cocktail
Benzonase
RNase H
Nuclease P1
Marker
Denaturing formaldehyde-MOPS 1% Agarose gel
Glycosylated molecules
Cy5 channel
EtBr channel
Transfer
Nylon membrane (ZetaProbe GT™)
Nitrocellulose membrane (Hybond C™)

Supplementary Figure 2. Comparison between silica-based column and TRIzol purification for the last clean-up step.

a. Schematics of the workflow.

b. Glycan detection in formaldehyde-denaturing agarose gels and blotted membranes. For nylon membrane, ZetaProbe GT™ from Bio-Rad™ was used. For nitrocellulose membrane, Hybond C™ from AmershamTM was used.

c. Glycan detection in gels and blotted membranes after RNase or DNase treatments. For nylon membrane, BrightStar-Plus™ from Invitrogen™ was used. For nitrocellulose membrane, Protran™ from Amersham™ was used.

d. Data represent other two replicates done in Fig. 2e.

e. Independent reproduction from a different lab's experiments for the effect of TRI Reagent and silica column purification on recovery. Final RNA purification in the silica column was performed at 60% EtOH. DNase I was used as an alternative for DNA degradation.

f. Glycan detection after various nucleases.

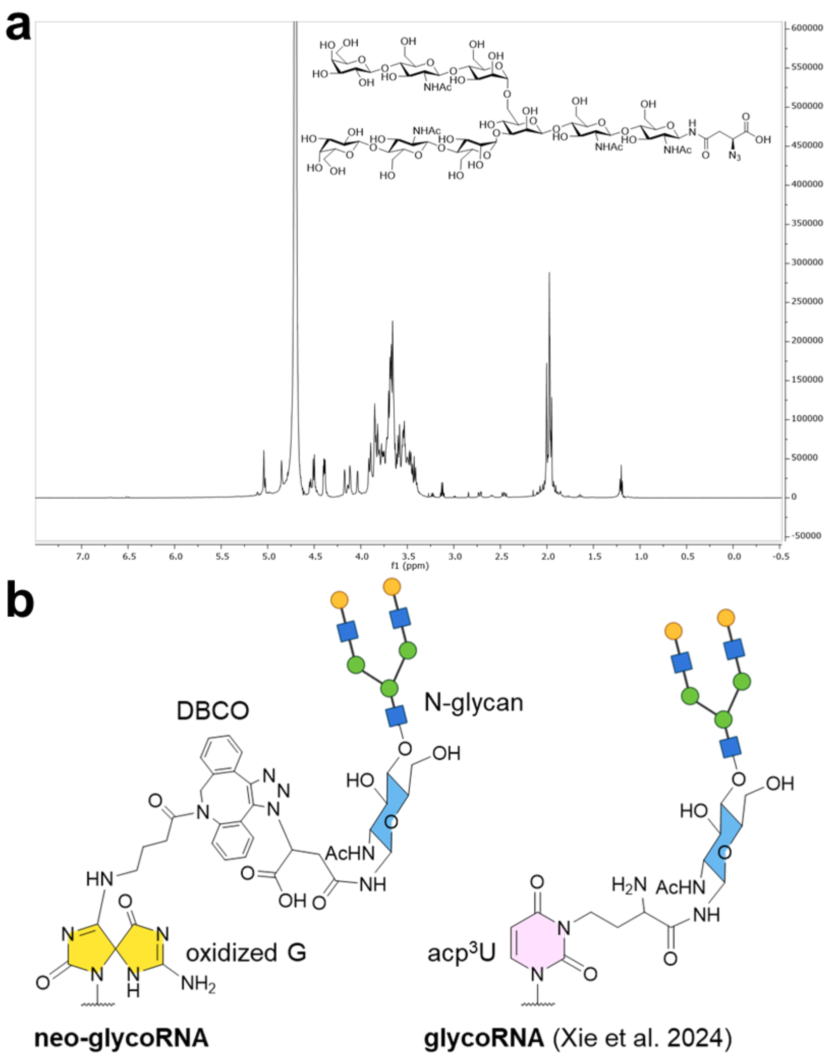

Supplementary Figure 3. Structure and supporting data for neo-glycoRNA.

a. Proton nuclear magnetic resonance demonstrating purity and quality of the azide-functionalized N-glycan.

b. Chemical structure of neo-glycoRNA and the comparison with reported glycoRNA. Abbreviations: G, guanosine; acp^{3}U, 3-(3-amino-3-carboxypropyl)uridine.

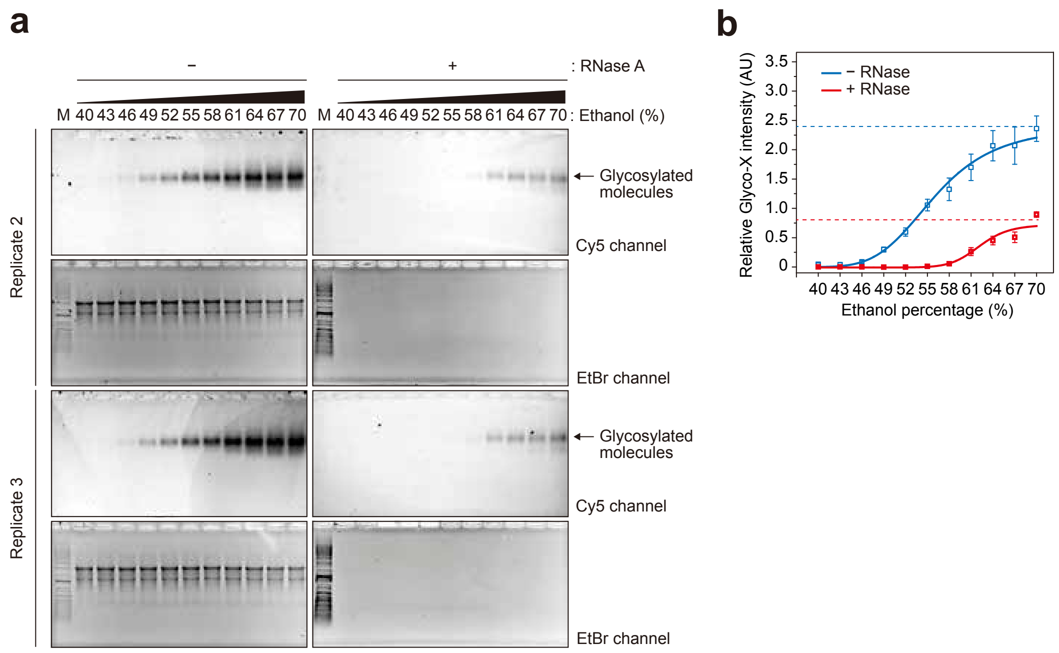

Supplementary Figure 4. Recovery of glycosylated molecules at various ethanol percentages.

a. Data represent other two replicates done in Fig. 4c.

b. Relative glycan intensity calculated from the data points in Fig. 3c and Supplementary Figure 3a. Error bars represent s.d.

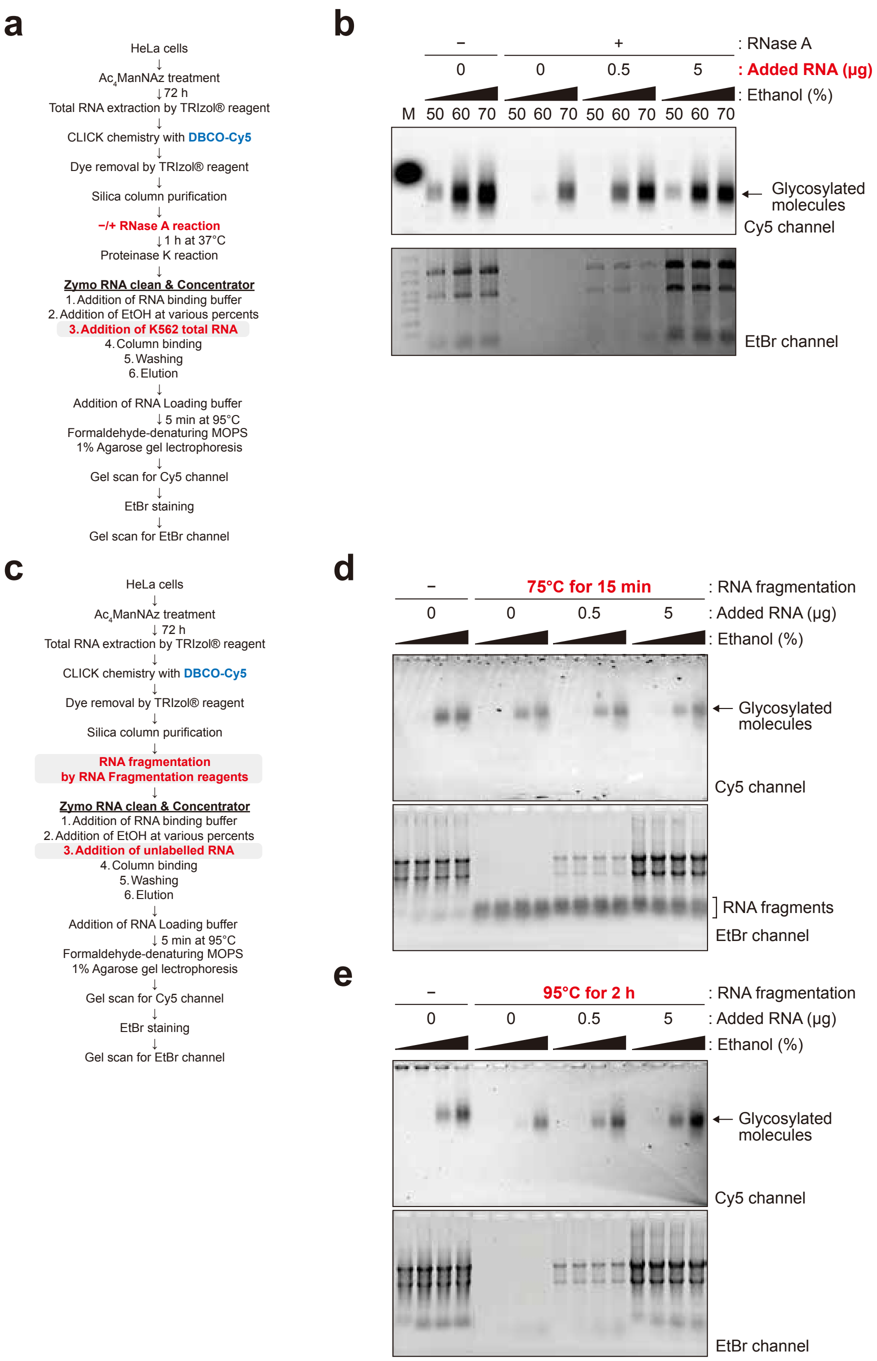

Supplementary Figure 5. Added RNA but not DNA improves the recovery rate of glycosylated molecules in RNA-depleted conditions.

a. Schematic for the experiment in Supplementary Figure 5b.

b. Data from the Li's experiment. Total RNA extracted from K562 cells was used as an alternative for added HeLa total RNA.

c. Schematic for experiments in Supplementary Figure 5d and 5e.

d. Glycan recovery by the added RNA in the mild RNA fragmentation condition. Partially fragmented RNAs are indicated in EtBr channel.

e. Glycan recovery by the added RNA in the complete RNA fragmentation condition.

Key resources table

Reagent or resource	Source	Identifier
**Chemicals, peptides, and recombinant proteins**		
Dulbecco’s Modified Eagle’s Medium (DMEM), high glucose with L-glutamine, sodium pyruvate and sodium bicarbonate	Welgene	Cat# LM001-17
Dulbecco’s Modified Eagle’s Medium (DMEM)	Gibco	Cat# 41965-039
Fetal bovine serum	Welgene	Cat# S001-01
Fetal bovine serum	Sigma-Aldrich	Cat# F7524
HyClone Characterized Fetal Bovine Serum	Cytiva	Cat# SH30071.03HI
Penicillin/streptomycin	Sigma-Aldrich	Cat# P4458
Dulbecco’s modified phosphate buffered saline (D-PBS), without calcium chloride and magnesium chloride	Welgene	Cat# LB001-02
Phosphate buffered saline (PBS)	In-house preparation	
Dimethyl sulfoxide (DMSO)	Sigma-Aldrich	Cat# 276855
Dimethyl sulfoxide (DMSO)	Merck	Cat# 1.0295.1000
N-azidoacetylmannosamine-tetraacylated ($Ac_4ManNAz$)	Sigma-Aldrich	Cat# 900917-50MG
N-azidoacetylmannosamine-tetraacylated ($Ac_4ManNAz$)	Custom synthesis by Synvenio	
TRIzol™ Reagent	Thermo Fisher Scientific	Cat# 15596018
TRI Reagent™ Solution	Thermo Fisher Scientific	Cat# AM9738
TRIzol™ LS Reagent	Thermo Fisher Scientific	Cat# 10296028
Chloroform	Sigma-Aldrich	Cat# C2432
TURBO™ DNase (2 U/μL)	Thermo Fisher Scientific	Cat# AM2238
DNase I (RNase free; 2U/μl)	Thermo Fisher Scientific	Cat# AM2222
RNase A (DNase and protease-free, 10 mg/mL)	Thermo Fisher Scientific	Cat# EN0531
PureLink™ RNase A (20 mg/mL)	Thermo Fisher Scientific	Cat# 12091021
Rapid PNGase F	New England Biolabs	Cat# P0710S
α2-3,6,8,9 Neuraminidase A	New England Biolabs	Cat# P0722S
Proteinase K, Recombinant, PCR grade	Roche	Cat# 3115879001

Proteinase K	Thermo Fischer Scientific	Cat# AM2548
RNase T1	Thermo Fischer Scientific	Cat# AM2283
RNase cocktail	Thermo Fischer Scientific	Cat# AM2286
Benzonase® Nuclease	Merck	Cat# E1014-25KU
RNase H	New England Biolabs	Cat# M0297L
Nuclease P1	New England Biolabs	Cat# M0660S
UltraPure™ Formamide	Thermo Fisher Scientific	Cat# 15515026
UltraPure™ 0.5M EDTA, pH 8.0	Thermo Fisher Scientific	Cat# 15575020
DEPC-Treated H_2O	Thermo Fisher Scientific	Cat# AM9920
Isopropanol, Optima LC/MS Grade	Fisher Scientific	Cat# A461-500
Ethyl alcohol, Pure	Sigma-Aldrich	Cat# E7023-1L
dibenzocyclooctyne-Cy5 (DBCO-Cy5)	Sigma-Aldrich	Cat# 777374-5MG
dibenzocyclooctyne-PEG4-biotin (DBCO-biotin)	Sigma-Aldrich	Cat# 760749-5MG
Linear acrylamide	Thermo Fisher Scientific	Cat# AM9520
SeaKem® LE Agarose	Lonza	Cat# 50004
NorthernMax® Denaturing Gel Buffer (10X)	Thermo Fisher Scientific	Cat# AM8676
NorthernMax® 10X Running Buffer	Thermo Fisher Scientific	Cat# AM8671
NorthernMax® Transfer Buffer	Thermo Fisher Scientific	Cat# AM8672
Odyssey Blocking Buffer (PBS)	Li-Cor Biosciences	Cat# 927-40000
IRDye 800CW Streptavidin	Li-Cor Biosciences	Cat# 926-32230
MOPS	Sigma-Aldrich	Cat# M1254
PBS Tablets	Gibco	Cat# 18912-014
TWEEN® 20	Sigma-Aldrich	Cat# P7949
Sodium Acetate·$3H_2O$	Merck	Cat# 1.06265.1000
EDTA	Sigma-Aldrich	Cat# EDS
37% Formaldehyde solution	Merck	Cat#1.04003.1000
UltraPure™ Ethidium Bromide, 10 mg/mL	Thermo Fisher Scientific	Cat# 15585011
Zeta-Probe® GT Membrane	Bio-Rad	Cat# 1620194
BrightStar™-Plus Positively Charged Nylon Membrane	Thermo Fisher Scientific	Cat# AM10102
Hybond-C nitrocellulose membrane	Cytiva	Cat# RPN303C
Amersham™ Protran® nitrocellulose membrane	Merck	Cat# GE10600001

Ambion® 10X RNA Fragmentation Reagent	Thermo Fisher Scientific	Cat# AM8740
**Critical commercial assays**		
NorthernMax™ Kit	Thermo Fisher Scientific	Cat# AM1940
RNA Clean and Concentrator 5	Zymo Research	Cat# R1013
**Experimental models: cell lines**		
HeLa	ATCC	Cat# ATCC-CCL-2
K562	ATCC	Cat# ATCC-CCL-24
